# Familial Hypercholesterolemia: Pitfalls and Challenges in Diagnosis and Treatment

**DOI:** 10.31083/j.rcm2408236

**Published:** 2023-08-17

**Authors:** Natalie Arnold, Wolfgang Koenig

**Affiliations:** ^1^Department of Cardiology, University Heart & Vascular Center Hamburg, University Medical Center Hamburg-Eppendorf, Hamburg, 20246 Hamburg, Germany; ^2^German Center for Cardiovascular Research (DZHK), Partner Site Hamburg/Kiel/Luebeck, Hamburg, Germany; ^3^Deutsches Herzzentrum München, Technische Universität München, 80636 Munich, Germany; ^4^German Centre for Cardiovascular Research (DZHK), Partner Site Munich Heart Alliance, Munich, Germany; ^5^Institute of Epidemiology and Medical Biometry, University of Ulm, 89081 Ulm, Germany

**Keywords:** familial hypercholesterolemia, atherosclerotic cardiovascular disease, lipid-lowering therapy

## Abstract

Familial hypercholesterolemia (FH), a condition, which is characterized by a life-long exposure to markedly elevated low-density lipoprotein (LDL) concentrations from birth, and it still remains underdiagnosed and undertreated, despite the fact that its heterogeneous form represents one of the commonest genetic disorders to date. Indeed, only 10% of all estimated affected individuals have been diagnosed worldwide and for the most of them diagnosis comes too late, when atherosclerotic cardiovascular disease (ASCVD) has already been developed. Undiagnosed and undertreated FH leads to accelerated ASCVD with a high rate of premature deaths. Recently, several novel treatment modalities have been introduced, especially for the management of severe hypercholesterolemia. Nonetheless, a substantial number of FH patients still do not achieve guideline-recommended LDL cholesterol target values. In the present review we will summarize and critically discuss pitfalls and challenges in successful diagnosis and treatment of FH.

## 1. Introduction

Being the most common genetic disorder to date [1], familial hypercholesterolemia (FH) remains vastly underdiagnosed and undertreated. Almost 60 years were needed from the first description of FH by the Norwegian physician Dr. Carl Müller [2] in the late 1930s until FH gained public health priority by the World Health Organization (WHO) in 1998 [3]. It took further 25 years until FH pediatric screening was recognized by the European Commission Public Health Best Practice Portal as one of the best practices in non-communicable disease prevention in 2022 [4]. Yet, challenges in FH have been unresolved for decades, despite the constantly growing scientific knowledge on its pathogenesis, recent development of novel therapeutics, and multiple efforts to overcome the existing gaps in FH care by, e.g., raising its awareness in the community. With these assumptions, it is not surprising that only 10% of all estimated affected individuals have been diagnosed worldwide [1]. Regrettably, only 2% of FH cases are diagnosed before the age of 18 years [1]. For most subjects, diagnosis occurs late in life, mostly at the age of about 45 years, often when atherosclerotic cardiovascular disease (ASCVD) has already developed [5, 6]. Thus, there is an unmet need for the identification of new index cases much earlier in their life course. Therefore, an integrated multidisciplinary approach including pediatricians, primary care physicians and clinicians in adult hospital settings is important to facilitate systematic FH screening in combination with reverse cascade screening of first degree relatives of FH patients [7, 8, 9, 10]. More importantly, even if the diagnosis seems to be certain, it does not always imply that the index patient is adequately treated. The European Atherosclerosis Society Familial Hypercholesterolemia Studies Collaboration (FHSC) global registry has impressively shown, that less than 3% of patients achieved the guideline-recommended low-density lipoprotein cholesterol (LDL-C) target values [5]. The present review summarizes our current knowledge about FH and critically discusses pitfalls and challenges in successful diagnosis and treatment of this genetic disorder.

## 2. Genetic and Phenotypic Heterogeneity of FH

As the name implies, FH represents an inherited disease, characterized by a life-long exposure to markedly elevated LDL-C concentrations from birth, thereby predisposing affected individuals to premature ASCVD.

For years, the “classical” form of FH has been recognized as an autosomal co-dominant monogenic condition, which is caused by variations of genes involved in LDL-C metabolism and clearance [11]. Among them, about 80% of genetic variants are caused by the mutation in the *LDLR* gene, encoding the LDL transmembrane receptor (LDL-R), which results in complete or partial loss of its function (so called “null” or “defective” LDL-R variants) [11, 12]. The remaining pathogenic variants are related to mutations within the genes encoding the apolipoprotein B (apoB) (*APOB*) (5–10%), with reduced binding of the apoB to the LDL-R or due to the gain-of-function mutation of proprotein convertase subtilisin/kexin 9 (*PCSK9*) (~3%), that lead to its overproduction [11, 12, 13, 14, 15]. In addition, the *APOE* gene represents another FH-causative gene, where single p.(Leu167del) mutation might occur in 1% to 2% of patients with FH phenotype and result in LDL-R downregulation [16]. In addition, there are also some other, sporadically occurring gene variants, e.g., within the genes encoding for signal-transducing adaptor protein family 1 (*STAP1*), patatin-like phospholipase-domain-containing family (*PNPLA5*) or some rare mutations, related to severe, recessive hypercholesterolemia, including LDLR adapter protein 1 (*LDLRAP1*), lysosomal acid lipase (*LIPA*) or ATP- binding cassette subfamily G member 5 (*ABCG5*) [11, 12, 17, 18]. Interestingly, some of these genes might also cause distinctive non-FH syndromes such as sitosterolemia (*ABCG5*), dysbetalipoproteinemia (*APOE*) or cholesteryl ester storage disease (*LIPA*) [19]. Despite a huge genetic heterogeneity (>2300 unique *LDLR* variants; >350 unique variants in *APOB* and >200 unique variants in *PCSK9*) [12, 17, 18, 20, 21] all above mentioned genetic variants have one common feature—they increase the LDL-C concentration dramatically, mainly by decreasing the clearance of LDL particles. In general, LDL-C concentration is dependent on whether the index patient is carrying mutations in both alleles (so called homozygous FH (HoFH)), causing severe hypercholesterolemia with LDL-C concentration mostly exceeding 400 mg/dL (>10 mmol/L) or if one allele is affected (heterozygous FH (HeFH)) with a LDL-C concentration mostly >190 mg/dL (>4.9 mmol/L) [18, 19, 20, 21, 22]. Although HoFH is a rather rare condition with an estimated worldwide prevalence of 1:300,000, HeFH represents the most common genetic disorder to date, affecting roughly 1 in 250–300 individuals in the population [23, 24, 25, 26]. Importantly, in certain patient groups, the prevalence of HeFH is even higher, e.g., 1 in 17 among patients with premature ASCVD [25]. Nonetheless, despite such a high prevalence of HeFH, this condition is still underdiagnosed. Based on an estimated prevalence, today we are dealing with the fact, that around 90% of affected subjects are still not aware of having FH [26, 27]. Interestingly, recent data have demonstrated that only about 60% of cardiologists and only 43% of general practitioners would diagnose FH correctly [28]. So, why has FH, for the diagnosis of which a simple LDL-C measurement is central, still such a low diagnostic rate [5]?

Typically, the likelihood for FH can be estimated primarily on the basis of the clinical phenotype. To date, there are several diagnostic algorithms available [29] (e.g., the Dutch Lipid Clinic Network (DLCN) Criteria, the Simon Broome (SB) system, the Make Early Diagnosis to Prevent Early Deaths (MEDPED) system, as well as the American Heart Association Agenda for FH criteria), which can be applied to diagnose FH, although DLCN, SB and MEDPED remain the most commonly used scores so far. All of these scores focus on LDL-C concentration, most of them also include personal and/or family history of premature coronary artery disease or dyslipidemia. Additionally, physical signs, all reflecting extravasal cholesterol deposits such as arcus cornea or bilateral xanthomas (within the Achilles tendons or within extensor tendon of the hand) might also be included (see Table 1 for the comparison between main existing algorithms).

**Table 1.** **Comparison between main diagnostic FH algorithms in adults**.

	Dutch Lipid Clinic Network	Simon Broome Register Group’s	MEDPED
Family history of hypercholesterolemia	I° relative with LDL-C >95th pctl (1 point)	I° or II° relative with TC >290 mg/dL (E)	Relative with confirmed FH diagnosis (I°/II°/III°)
	Children (<18 y old) with LDL-C >95th pctl (2 points)		
Elevated LDL-C (untreated)	≥330 mg/dL (≥8.5 mmol/L) (8 points)	≥190 mg/dL (≥4.9 mmol/L) (A)	Relative I°/II°/III°/general population
	250–329 mg/dL (6.5–8.4 mmol/L) (5 points)		<20 y: 220/230/240/270 mg/dL / 5.7/5.9/6.2/7.0 mmol/L
	190–249 mg/dL (5.0–6.4 mmol/L) (3 points)		20–29 y: 240/250/260/290 mg/dL / 6.2/6.5/6.7/7.5 mmol/L
	155–189 mg/dL (4.0–4.9 mmol/L) (1 point)		30–39 y: 270/280/290/340 mg/dL / 7.0/7.2/7.5/8.8 mmol/L
			≥40 y: 290/300/310/360 mg/dL / 7.5/7.8/8.0/9.3 mmol/L
Family history of premature coronary artery disease	I° relative with known premature coronary and/or vascular disease (♂ <55 y old, ♀ <60 y old) (1 point)	I° relative with MI (<60 y old) or II° relative with MI (<50 y old) (D)	-
Family history of tendon xanthomas	I° relative with tendinous xanthomata and/or arcus cornealis (2 points)	I° relative with xanthomas (B)	-
Personal history	- Patients with premature coronary artery disease (♂ <55 y old, ♀ <60 y old) (2 points)	-	-
	- Patients with premature cerebral or peripheral vascular disease (♂ <55 y old, ♀ <60 y old) (1 point)		
Physical examination	Tendinous xanthomata (6 points)	Xanthomas in the proband (B)	-
	Arcus cornealis <45 y old (4 points)		
Genetic analysis	Mutation in the *LDLR*, *APOB* or *PCSK9 *gene (8 points)	Mutation in the *LDLR*, *APOB* or *PCSK9* gene (C)	-
Diagnosis	Unlikely FH: <3	Possible FH: A + D or A and E	FH is diagnosed if LDL-C exceed the cut point
	Possible FH: 3–5	Definitive FH: A + B or C	
	Probable FH: 6–8		
	Definite FH: >8		

FH, familial hypercholesterolemia; MEDPED, “Make Early Diagnosis to Prevent Early Death”; LDL-C, low density lipoprotein cholesterol; pctl, percentile; TC, total cholesterol; y, year; MI, myocardial infarction; *LDLR*, low-density lipoprotein receptor; *APOB*, apolipoprotein B; *PCSK9*, proprotein convertase subtilisin/kexin 9.

Subsequent genetic testing with confirmation of a pathogenic mutation in the FH causative gene would provide diagnostic certainty, although this is not essential for the diagnosis itself. Nonetheless, identification of a positive mutation is important for the initiation of cascade screening to detect FH in other family members as well as for the early initiation of lipid-lowering treatment (LLT) and might be implicated in the choice of treatment in FH. Moreover, genetic confirmation of FH is extremely helpful in identifying subjects with the highest risk for ASCVD, since the presence of a “classic” FH mutation in subjects with LDL-C levels >190 mg/dL (>4.9 mmol/L) results in a 3.7-fold increased coronary heart disease (CHD) risk, compared to subjects with equally elevated LDL-C but not carrying a genetic variant. This risk increases further, even up to 20-fold, by comparison to normolipidemic individuals [30].

Nonetheless, optimal screening strategies to identify index patients on the population level have not yet been determined. The commonly used diagnostic tools for the severe hypercholesterolemic phenotype in the clinical setting rely on already manifested clinical symptoms/diseases, thereby having only limited utility in primary care for the early (asymptomatic) FH case-finding. Furthermore, xanthomas and corneal arcus can be detected only in <15% and 30%, respectively of HeFH patients, as has been shown by the Spanish Familial Hypercholesterolemia Cohort study [31], probably due to earlier and much broader introduction of LLT. Ongoing treatment might also mask an initially increased “untreated” LDL-C concentration, showing on average lower LDL-C than perhaps expected, although first attempts have been undertaken to calculate pre-treated LDL-C concentrations using information on the dose and type of treatment [32].

In line with all of the above mentioned findings are the results of several studies, showing significant variability in the accuracy of clinical algorithms in those with genetically confirmed FH [33, 34]. One extreme example represents the analysis by Mohammadnia *et al*. [33], who demonstrated that the sensitivity of currently available scores for the clinical FH diagnosis in subjects, positive for FH gene mutations is only modest, being 9% for DLCN ≥6, 17% for SB and 31% for MEDPED. Within another clinical cohort, analyzing genomic sequence and clinical data from 50,726 individuals from the Geisinger Health System, showed that only 24% out of 215 carriers of a FH variant met criteria for definite or probable clinical FH. More importantly, 44% were classified as ‘unlikely FH’ [35].

On the other hand, recent data from the large-scale population-based studies using next-generation DNA sequencing have found that on a molecular-genetic level FH is more complex than previously assumed. Importantly, the majority of individuals who meet clinical FH criteria do not possess a causative gene defect within the main, “classical” FH genes [30, 35, 36, 37, 38, 39], although the prevalence of identified mutations might vary significantly depending of the applied clinical criteria or the clinical setting (from the general population to the tertiary care lipid clinics). For instance, data from one large study, including ~20,000 individuals from the general population have demonstrated that in subjects with LDL-C ≥190 mg/dL (≥4.9 mmol/L) the mutation within one of three genes causative for FH (*LDLR*, *APOB*, and *PCSK9*) could be found only in 1.7% of participants [30]. Similar results have been obtained by Abul-Husn *et al*. [35], using exome sequencing and electronic health records of 50,726 individuals and showing that classical FH variants explain only 2.5% of severe hypercholesterolemia. Among 48,741 individuals of the UK Biobank exome sequencing cohort, only 0.57% had a monogenic FH-associated variant [37]. Data from the Dutch FH cohort showed that in 85% of cases no genetic confirmation of FH could be found [38].

Yet, the prevalence of causative FH mutation among patients with suspected FH, referred to the tertiary care lipid clinics, seems to be higher. Genetic confirmation might be found among up to two-thirds of such patients and even ~90% in those with untreated LDL-C levels >310 mg/dL (>8 mmol/L) [39]. Otherwise, there is also clear evidence that finding a rare gene variant does not necessarily express itself as phenotypical FH (i.e., genetically verified FH without clinical FH), since normal or only moderately elevated LDL-C levels can be documented in patients with an identified causal variant of FH [40].

Thus, a substantial number of patients with clinical FH phenotype (both very high LDL-C levels and positive family history) but without monogenic mutation would suggest polygenic causes of FH, where small but cumulative effects of several LDL-C raising alleles can cause the LDL-C increase up to the same range as that caused by the three primary FH-causing genes [12, 17, 18]. Indeed, up to 100 polymorphic loci might contribute to polygenic susceptibility to elevated LDL-C [12, 41, 42]. In addition, presence of negative genetic test results might also imply presence of causal mutations within the still unidentified genes, so called genetically undefined hypercholesterolemia [43].

Interestingly, some substantial differences between monogenic and polygenic FH might exist with regard to the clinical presentation, cardiovascular risk and responsiveness to therapy (for comprehensive review please see Ref. [43, 44]). For instance, it could be shown that subjects with monogenic FH not only have statistically higher LDL-C concentration (typically by ~>40 mg/dL (>1 mmol/L)), but also tended to develop more severe atherosclerosis than subjects with polygenic FH [37, 45, 46, 47]. Moreover, cardiovascular risk, related to monogenic FH might be also higher, than polygenic FH-related risk [37, 45, 46, 47]. Pathophysiologically, polygenic origin of FH would, however, result in LDL-C overproduction, rather than in catabolic defects, as seen among their monogenic forms and therefore would probably show a better response to treatment. Indeed, there is first evidence that lipid-lowering therapy is more effective in subjects with a polygenic background, compared to subjects with canonical FH mutations [46, 48]. Although clinical presentation of polygenic FH seems to be less severe than its monogenic form, cardiovascular risk is still very high in these patients compared to control normolipidemic subjects.

Taken together, there is not only a clear mismatch between clinical and genetic diagnosis of FH, but also significant differences in its genetic background (mono- versus polygenic), that might significantly complicate the recognition of FH in daily practice. Nonetheless, a simple combination of untreated LDL-C >190 mg/dL (>4.9 mmol/L) in adults and the presence of premature CAD in index patients or her/his first-degree relatives would dramatically raise the suspicion of FH in the clinical routine, thereby performing a simple practical approach for better identification of FH patients.

## 3. Lipoprotein(a) in FH

Another challenge in the diagnosis of FH is the LDL-C measurement. Conventional assays for LDL-C determination quantify a composite of atherogenic cholesterol, which is attributable not only to LDL-C, but also to lipoprotein(a)-cholesterol (Lp(a)-C) due to their overlapping densities. Lp(a) represents a genetically determined highly atherogenic LDL-like particle, which has been considered a novel risk factor for ASCVD and aortic stenosis [49]. Early studies have shown that subjects with diagnosed FH had higher levels of Lp(a) [50, 51], compared to non-affected individuals, thereby assuming that FH might lead to an increase in Lp(a). Indeed, elevated Lp(a) is present in 30–50% of FH patients [52]. However, the role of Lp(a) in FH seems to be much more complex. Recent data suggest that increased Lp(a) levels might, at least in part, mimic the clinical diagnosis of FH, probably due to the Lp(a)-C component within “overall” LDL-C quantification [53, 54, 55, 56]. For instance, among 46,200 individuals from the Copenhagen General Population Study, about 25% of individuals with clinical FH were diagnosed because of high Lp(a) levels [54]. A series of studies, conducted within the last 2–3 years provided very consistent results, showing that the Lp(a)-C content in LDL-C in subjects with suspected HeFH can lead to reclassification of clinical FH status [54, 55, 56, 57, 58]. For instance, Hedegaard *et al*. [58] recalculated the DLCN scores after adjusting for the contribution of Lp(a) to LDL-C values and found that 16.6% of patients fell into a lower DLCN category. Furthermore, two other studies showed that up to 10 patients with clinical suspicion of FH could be down-classified to the category “unlikely FH” using Lp(a)-corrected LDL-C [55, 56], thereby avoiding unnecessary genetic analysis for FH.

Unfortunately, how LDL-C should be corrected for its Lp(a)-C content is not entirely clear, especially taking into account a possible variation of Lp(a)-C relative to its mass, which might vary from 6% to 60% [59, 60, 61]. Most importantly, by applying the “wrong” correction one could also miss mutation positive subjects. Nonetheless, taking into account the fact that all diagnostic FH algorithms rely on plasma LDL-C level, an interrelationship between Lp(a)-C and “true” LDL-C should not be underestimated, especially in those with LDL-C levels that are borderline consistent with HeFH. Although several issues still have to be clarified on the role of Lp(a) in FH, it is clear, having both FH and high Lp(a) values >50 mg/dL results in an extremely high risk of myocardial infarction in the general population [51, 54, 55].

## 4. Current Treatment Options in Patients with FH

**Fig. 1.**
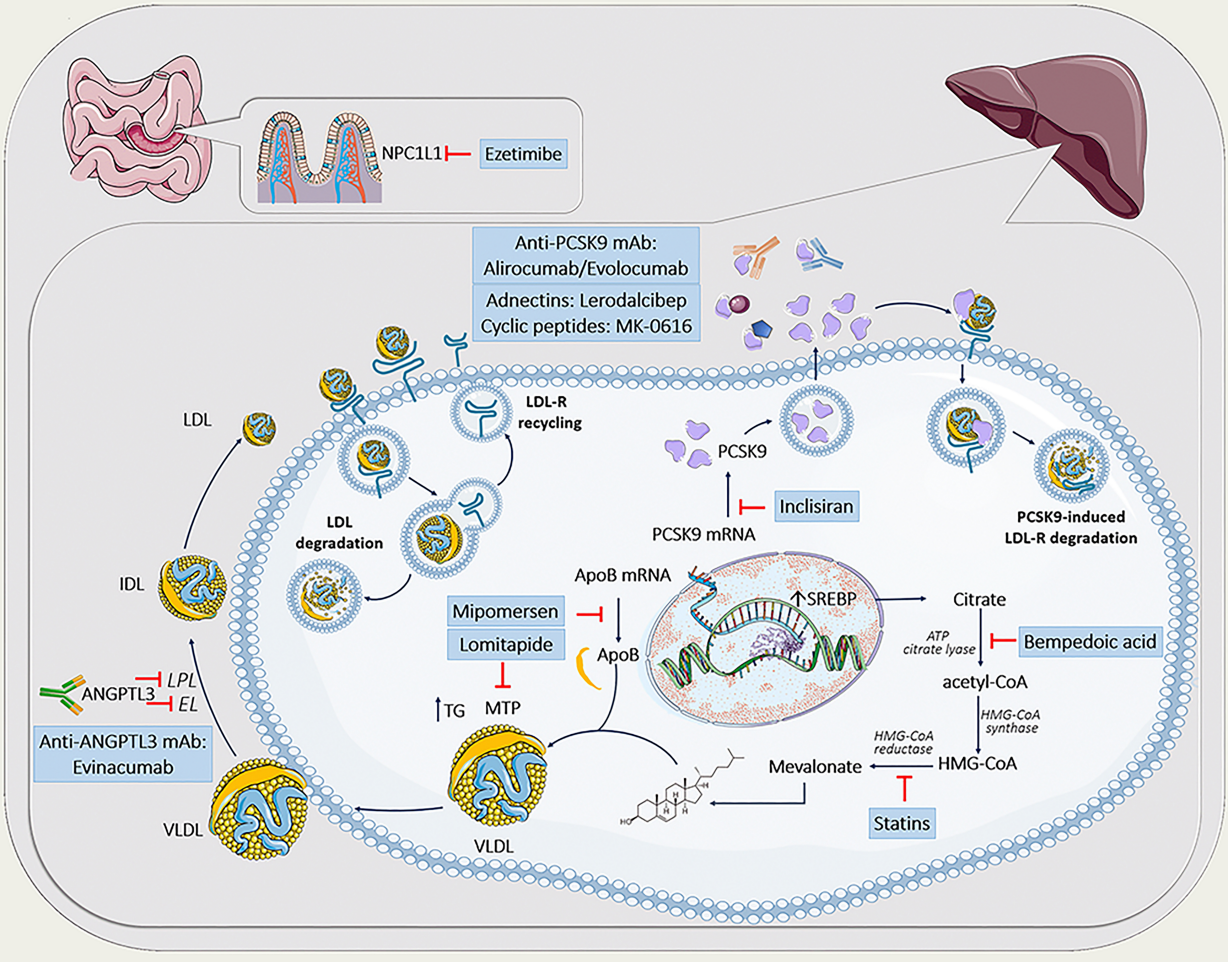
**Lipid-lowering agents in the treatment of familial hypercholesterolemia**. The figure was partly generated using Servier Medical Art, provided by Servier, licensed under a Creative Commons Attribution 3.0 unported license. ANGPTL3, angiopoietin-like protein 3; apoB, apolipoprotein B; ATP, adenosine triphosphate; CETP, cholesteryl ester transfer protein; EL, endothelial lipase; HDL, high density lipoprotein; HMG-CoA, 3-hydroxy-3-methylglutaryl coenzyme A; IDL, intermediate-density lipoprotein; LDL, low-density lipoprotein; LDL-R, low-density lipoprotein receptor; LPL, lipoprotein lipase; mAb, monoclonal antibodies; MTP, microsomal triglyceride transfer protein; NPC1L1, Niemann-Pick C1-like 1 protein; PCSK9, proprotein convertase subtilisin kexin type 9; SREBP, sterol regulatory element-binding protein; mRNA, messenger RNA; TG, triglycerides; VLDL, very-low-density lipoprotein.

Being mostly asymptomatic, lifelong exposure to elevated LDL-C, if untreated, leads to premature development and accelerated progression of ASCVD. Thus, early introduction of therapeutic interventions is essential for improved prognosis of patients with FH. Currently, the European Society of Cardiology/European Atherosclerosis Society (ESC/EAS) 2019 guidelines recommend at least 50% LDL-C reduction and LDL-C target <70 mg/dL (<1.8 mmol/L) for FH patients without cardiovascular risk factors and <55 mg/dL (<1.4 mmol/L)/for FH patients with another major cardiovascular risk factor or clinical ASCVD (“very high-risk”) [62]. However, achieving the LDL-C recommended target values still seems to be very challenging [5], despite the availability of a variety of lipid-lowering drugs, which can be used in clinical routine to treat FH successfully. Fig. 1 depicts sites of action of various lipid lowering agents in FH.

### 4.1 Statins, Ezetimibe and Bempedoic Acid

For years, statins (alone or in combination) represent a cost-effective first-line therapy in subjects with FH, particularly in heterozygous patients [63, 64]. In general, high-potency statins are capable of lowering LDL-C by 50% to 60% as monotherapy and even by 65 to 70% if combined, e.g., with ezetimibe, a Niemann-Pick C1-like protein inhibitor [65, 66]. Both compounds, although acting differentially (statins by decreasing cholesterol production via selective inhibition of 3-hydroxy-3-methylglutaryl coenzyme A (HMG-CoA) reductase; ezetimibe by blocking cholesterol uptake from the jejunum) result in a compensatory increase in LDL-R and subsequently enhanced LDL-C clearance. So, in subjects with FH, having a dysfunctional LDL-R, LDL-C lowering effects of this standard LLT might be only modest [63]. In addition, the presence of increased Lp(a) might also influence the LDL-C lowering ability of statins, particularly in those with smaller apo(a) isoforms, either by decreasing an apparent response to LDL-C lowering or even increasing the LDL-C concentration [67, 68]. Nonetheless, statins (alone or in combination with ezetimibe) demonstrated a significant reduction of future ASCVD events even in subjects with LDL-R defective forms [69, 70, 71, 72, 73].

More recently, bempedoic acid (BA), another inhibitor of intracellular cholesterol biosynthesis, has been introduced in the clinical setting. It acts as an inhibitor of adenosine triphosphate (ATP) citrate lyase, a hepatic enzyme that works upstream of HMG-CoA reductase with subsequent upregulation of LDL-R activity, similar to statins [74]. Pooled analysis of 112 patients with a clinical phenotype of HeFH, participating in phase 3 trials (CLEAR Harmony and CLEAR wisdom) showed a mean LDL-C reduction of 22.3% by BA, applied as an adjunct or alternatively to currently existing LLT [75]. However, whether BA would also reduce LDL-C in HoFH has not been investigated so far. But, based on the mechanism of action of BA, which is similar to statins, it is possible that patients with residual LDL-R activity will respond to it as well.

### 4.2 PCSK9 Inhibition

The development of PCSK9 inhibitors has provided an additional therapeutic tool to control LDL-C in FH patients with residual LDL-receptor activity. In 2003, a novel gain-of-function mutation within the *PCSK9* gene, contributing to a phenotype with markedly elevated LDL-C levels and premature ASCVD, had been identified in patients with severe hypercholesterolemia [15]. PCSK9 decreases recycling and increases degradation of the LDL-R (Fig. 1). To date, there are only two approved modalities to inhibit PCSK9 activity. Alirocumab and evolocumab are fully human monoclonal antibodies (mAbs) targeted against PCSK9, whereas inclisiran represents a first-in-class cholesterol-lowering small interfering ribonucleic acid (siRNA), targeting PCSK9 messenger RNA (mRNA) in hepatocytes. In contrast to anti-PCSK9 mAbs, inclisiran inactivates PCSK9 by inhibition of its hepatic synthesis [76].

So far, there are several trials that have assessed the efficacy of anti-PCSK9 mAbs in HeFH, including ODYSSEY FH I/II, ODYSSEY HIGH FH, RUTHERFORD-2, as well as HAUSER-RCT, all reporting meaningful LDL-C reductions by alirocumab or evolocumab between 45 and 65% [77, 78, 79, 80, 81]. However, in HoFH and LDL-R-negative mutations, anti-PCSK9 mAbs would probably only be mildly effective or even fail to lower LDL-C [82, 83, 84]. So, current guidelines also recommend the use of PCSK9 inhibitors to treat homozygous FH patients except those with confirmed negative/negative LDLR mutations.

Inclisiran might also be a promising option to treat FH patients, showing a mean LDL-C reduction of 40% in HeFH subjects within the ORION-9 trial [85]. Also, in the ORION-2 trial, an open-label pilot study in 5 HoFH patients receiving high-intensity statin plus ezetimibe, inclisiran exhibited similar LDL-C lowering, compared to those, observed for anti PCSK9-mAb, although with a longer effect duration [86]. A phase 3 study of inclisiran in HoFH (NCT03851705), including 56 patients with HoFH and LDL-C >130 mg/dL (>3.4 mmol/L), despite a maximally tolerated lipid-lowering background therapy is currently ongoing and results are expected in the near future.

There are also several emerging PCSK9 inhibitors such as Lerodalcibep (recombinant fusion protein, consisting of a PCSK9-binding domain (adnectin)) or MK-0616 (synthetic cyclic peptide, being a first orally bioavailable PCSK9 inhibitor) which are currently being tested in FH patients (Lerodalcibep: NCT04034485 for HoFH and NCT04797104 for HeFH; MK-0616: NCT05261126).

### 4.3 Novel LDL-R Independent Therapeutics

Despite a large armamentarium of potent lipid-lowering medication, including statins, ezetimibe, BA and PCSK9 inhibitors, which demonstrate a cumulative ability to lower LDL-C >85% [62, 87]. LDL-C levels remain far above the target LDL-C in patients with severe refractory HeFH and especially in HoFH subjects, where a dysfunctional LDL-R represents a major pitfall of successful LDL-C lowering. In other words, in HoFH patients with extremely high LDL-C concentration (>400 mg/dL (>10.4 mmol/L)) guideline-recommended LDL-C (<70/55 mg/dL) (<1.8/1.4 mmol/L) would be hardly achieved by the above mentioned therapy [88, 89]. So, additional therapeutic interventions that work independently of the LDL-R pathway are urgently needed. To such novel LLT, which lower LDL-C via LDL-R independent mechanisms belong to inhibitors of apoB/VLDL secretion as well as ANGPTL3-inhibitors (Fig. 1).

#### 4.3.1 Inhibitors of apoB/ VLDL Secretion

Being a cellular protein, responsible for the transport of neutral lipids between membrane vesicles, microsomal triglyceride transfer protein (MTP) plays a pivotal role in apoB secretion [90]. Lomitapide, the first MTP inhibitor, exclusively used for patients with HoFH with or without lipid apheresis, reduce LDL-C concentration by 40–50% primarily via decreased VLDL and apoB secretion [91].

Another inhibitor of apoB/VLDL secretion is Mipomersen, an antisense oligonucleotide (ASO) targeted to the APOB mRNA [90], having a potential to lower LDL-C by ~25% in patients with HoFH [92, 93]. It is approved for HoFH patients in the U.S. but EMA refused mipomersen marketing authorization.

Unfortunately, both therapeutic compounds have strong gastrointestinal side effects and might significantly increase hepatic fat deposition, leading to hepatosteatosis, thereby limiting their use in patients with FH [90, 93].

#### 4.3.2 ANGPTL3-Inhibitors

Angiopoietin-like 3 protein (ANGPTL3) is an endogenous inhibitor of endothelial and lipoprotein lipase, the latter representing a key enzyme involved in the removal of triglycerides rich lipoproteins from the circulation [94, 95]. The discovery of ANGPTL3 as a potential treatment target came from Genome-wide association study (GWAS), where subjects with a loss-of-function mutation within the ANGPTL3 gene demonstrated a 41% lower risk of ASCVD due to life-long low levels of both LDL-C and triglycerides [96]. Although the role of ANGPTL3 in lowering LDL-C is still not completely understood one might suggest, that ANGPTL3 inhibition enhances fractional catabolic rate of large VLDL thereby reducing LDL-C through faster clearance of their remnants by non-LDL-R-mediated pathways [97].

Evinacumab, the first available ANGPTL3 inhibitor is a human monoclonal antibody for ANGPTL3, which has been approved for the treatment of patients with HoFH [98]. Approximately 50% LDL-C reduction under evinacumab therapy has been demonstrated in HoFH subjects and those with refractory hypercholesterolemia [99, 100]. More importantly, even in patients with null/null variants in the LDL-R a 43% reduction in LDL-C has been seen, indicating LDL-R independent pathway of lipid lowering.

Finally, a first siRNA targeting ANGPTL3 mRNA is also under development (ARO-ANG3), demonstrating an approximately 40% LDL-C reduction in a phase I trial [101]. ARO-ANG3 is currently being tested in phase 2 trials in patients with HoFH (NCT05217667) or mixed dyslipidemia (NCT04832971).

#### 4.3.3 Cholesteryl Ester Transfer Protein (CETP) Inhibition

Since FH patients might have dysfunctional high density lipoproteins (HDL), resulting in defective reverse cholesterol transport (RCT) and subsequent increase in cholesteryl ester transfer protein (CETP) in the circulation [102], inhibition of CETP, a hydrophobic glycoprotein that promotes the transfer of cholesteryl ester and triglyceride between all lipoproteins, might represent another possible target in FH. Although initial studies on CETP inhibitors were rather disappointing [103], the data on the newest CETP inhibitor obicetrapib seems to be more promising, achieving reductions in LDL-C up to 50% [104]. Currently, obicetrapib has been tested within the phase III study (BROOKLYN) (NCT05425745) in patients with HeFH on top of maximum tolerated lipid-modifying therapies. More interestingly, obicetrapib might also lower Lp(a) level by approximately 50%. However, the pathophysiological mechanism responsible for such profound Lp(a) lowering is still poorly understood.

Taken together, our therapeutic armamentarium to combat FH increased significantly during the last years allowing us to prevent/reduce future cardiovascular events more successfully [105]. However, despite such significant improvement in the pharmacologic intervention, initiation of lipoprotein apheresis (LA) in addition to existing drug therapy is foundational for a still substantial proportion of FH patients (HoFH or with increased Lp(a) level) and might represent the only way to attain the guideline-recommended LDL-C targets [89]. On the other hand, there is clear evidence that novel FH therapeutics might significantly reduce the need for LA [89].

## 5. Conclusions

Familial hypercholesterolemia remains vastly underdiagnosed and as a consequence, undertreated, resulting in a missed opportunity to delay or even prevent clinical manifestations of atherosclerosis. Early detection of FH, including wide-spread pediatric screening programs, as well as increased medical community awareness of FH should become a priority worldwide to improve the low diagnostic rates of FH. A further major challenge in FH represents its definition, since recent research has reshaped our understanding of the pathogenesis of FH, indicating that FH is not an exclusively monogenic disorder. Finally, significant treatment gaps are still existing, demanding not only novel therapeutics, but also their broad accessibility. Although current efforts in FH management are still hampered, integrated implementation of strategies worldwide to identify, diagnose and successfully treat FH patients would undoubtedly lead to a significant reduction of FH burden.
